# Genome of Emerging Norovirus GII.17, United States, 2014

**DOI:** 10.3201/eid2108.150652

**Published:** 2015-08

**Authors:** Gabriel I. Parra, Kim Y. Green

**Affiliations:** National Institutes of Health, Bethesda, Maryland, USA

**Keywords:** norovirus, diarrhea, GII.17, viruses, genome, China, United States

## Abstract

To determine whether the norovirus strain GII.17 recently detected in Maryland, USA, (Hu/GII.17/Gaithersburg/2014/US) is spreading globally, we characterized the genome. High similarity with the norovirus GII.17 that caused recent outbreaks in Asia indicates that the same strain was present in the United States during the 2014–15 norovirus season (winter).

Noroviruses are major pathogens associated with acute gastroenteritis among persons in all age groups. In developing countries, noroviruses are responsible for an estimated 200,000 deaths per year among children <5 years of age (*1*). These viruses characteristically cause outbreaks in partially enclosed settings such as schools, childcare centers, nursing homes, military facilities, and cruise ships (*2*).

Noroviruses possess a genome of single-stranded positive-sense RNA that is organized into 3 open reading frames (ORFs). ORF1 encodes the nonstructural proteins required for replication, including the RNA-dependent RNA polymerase (RdRp); ORF2 encodes the major capsid protein (viral protein [VP] 1); and ORF3 encodes the minor capsid protein (VP2) (*2*). Noroviruses are genetically diverse and are divided into 6 genogroups (GI–GVI) and ≈30 genotypes according to comparison of VP1 sequences. The fact that patients have been sequentially infected with distinct strains suggests a lack of cross-protection among genotypes (*3*–*5*). The current norovirus classification system uses a dual nomenclature based on differences in the RdRp (polymerase or P genotype) and the VP1 region (capsid or G genotype) (*6*). Because noroviruses are prone to recombine within the ORF1/ORF2 junction, strains with different combinations of P and G genotypes can be detected in nature (*7*).

Although several norovirus strains circulate, for ≈2 decades, GII.4 has been the predominant genotype infecting humans. It has been proposed that GII.4 strains successfully persist and infect humans by the periodic emergence of new GII.4 variants that escape from herd immunity developed against previous variants (*8*). Recently, increased detection of GII.17 as the predominant outbreak strain in China has been reported (*9*). In this study, we characterized the genome of a norovirus GII.17 strain recently detected in Maryland, USA, to determine whether the same GII.17 virus is spreading globally.

## The Study

On November 25, 2014, acute gastroenteritis developed in a 3-year-old child in Gaithersburg, Maryland, USA. The child’s parent gave informed consent for the child’s enrollment in National Institutes of Health (NIH) clinical study NCT01306084. No other family member became ill, and no one in the family had a history of recent travel. A fecal sample was collected from the child and tested for norovirus by reverse transcription PCR (RT-PCR) by using generic primers that annealed to the polymerase region. Results were confirmed by nucleotide sequencing, and the P genotype was assigned by using the Norovirus Genotyping Tool (http://www.rivm.nl/mpf/norovirus/typingtool) (*10*). The capsid region of the sample was assigned to GII.17, and the polymerase region was classified as “unknown genotype.” To further assess the identity of the virus, we amplified the complete genome by using RT-PCR and primers selective for the conserved 3′ and 5′ end regions; we sequenced it by using an Ion Torrent platform (Life Technologies, Carlsbad, CA, USA). The sequence of the virus (designated Hu/GII.17/Gaithersburg/2014/US) was deposited into GenBank under accession no. KR083017.

**Figure 1:**
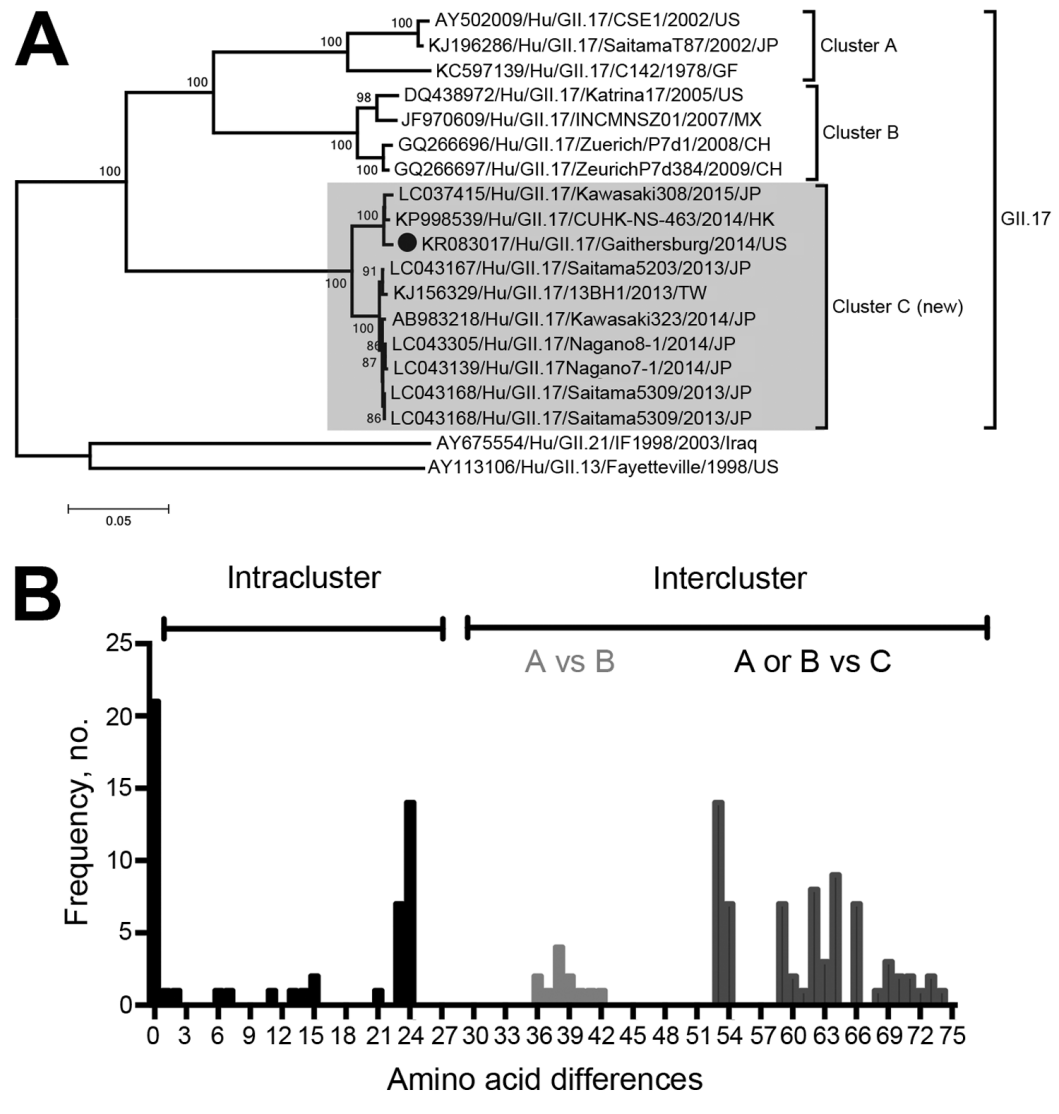
Relationship of major capsid protein (viral protein [VP] 1) of norovirus strain Hu/GII.17/Gaithersburg/2014/US with other GII.17 noroviruses. A) Phylogenetic tree of the Hu/GII.17/Gaithersburg/2014/US VP1 region showing comparison with those of GII.17 norovirus strains available in public genetic databases. Phylogenetic analyses were conducted by using MEGA version 6 (*11*), neighbor-joining as the algorithm for reconstruction, and Tamura-Nei as the model of substitution. Bootstrap (500 replicates) analysis was used for the statistical support of the tree; values >70% are shown. The Hu/GII.17/Gaithersburg/2014/US strain is indicated by a black circle. For each strain, the GenBank accession number/name/year of detection/country is shown. Gray shading indicates GII.17 cluster C strains. Scale bar indicates nucleotide substitutions per site. B) Amino acid differences among the GII.17 strains.

A BLAST search (http://blast.ncbi.nlm.nih.gov/Blast.cgi) identified 9 strains (all recently detected in countries from Asia) as having the highest similarity to Hu/GII.17/Gaithersburg/2014/US. A phylogenetic analysis of all GII.17 strains with full-length (or nearly full-length) capsid regions deposited in the GenBank database revealed the presence of 3 distinct clusters (A–C); the Hu/GII.17/Gaithersburg/2014/US strain clustered with strains recently detected in Taiwan, Hong Kong, and Japan and not with the GII.17 strain detected before 2009 (Figure 1). The 3 clusters differed by >36 aa substitutions; the greatest divergence was found for cluster C (Figure 1). Sequence analysis of the VP1 of strains representing each cluster showed a number of amino acid substitutions unique for each cluster (62/543). Three deletions (residues 295, 296, and 384) and 1 insertion (Asp344) were present only in the strains from cluster C. Of note, the Hu/GII.17/Gaithersburg/2014/US strain presented 25 aa substitutions, compared with the other cluster C strains, and contained 2 unique insertions (Asp380 and Asp396) only present in 2 GII.7 strains from cluster C (Hu/GII.17/CUHK-NS-463/2014/HK and Hu/GII.17/Kawasaki308/2015/JP). Molecular modeling of the GII.17 capsid showed that most of the substitutions (43/62) mapped onto the surface of the VP1. The 2 deletions (295–296) of the novel GII.17 (cluster C) mapped into 1 of the major epitopes (epitope A) described for GII.4 norovirus, and the remaining insertions and deletions also mapped near residues involved in major epitopes (*8*) (Technical Appendix). These modifications in the capsid of the new GII.17 cluster C strains might confer new antigenic or biological characteristics that would provide evolutionary advantages for infection, rapid spread, or both.

**Figure 2:**
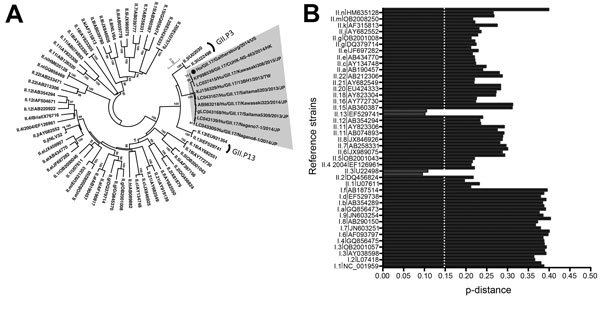
Relationship of the RNA-dependent RNA polymerase (RdRp) region of norovirus strain Hu/GII.17/Gaithersburg/2014/US to that of other noroviruses. A) Phylogenetic tree of the RdRp region (nt 4526–5116) from novel GII.17 strains (cluster C) and reference strains from each genotype described (*6*). Phylogenetic analyses were conducted by using MEGA version 6 (*11*), neighbor-joining as the algorithm for reconstruction, and Tamura-Nei as the model of substitution. Bootstrap (500 replicates) analysis was used for the statistical support of the tree. Gray shading indicates GII.17 cluster C strains, and Hu/GII.17/Gaithersburg/2014/US is indicated by a black circle. Strains from genotypes GII.P3 and GII.P13 are indicated by brackets. B) Distances (p-distances) among the Hu/GII.17/Gaithersburg/2014/US strain and reference strains. Values compared with reference strains from genotypes GII.P3 and GII.P13 are shaded. Values considered cut off for genotype designation are indicated with a dotted line (*6*,*12*).

Analysis of the RdRp region showed that the Hu/GII.17/Gaithersburg/2014/US strain clustered between GII.3 and GII.13 strains and had genetic distances of ≈0.102 and ≈0.103, respectively (Figure 2). Although the RdRp from the Hu/GII.17/Gaithersburg/2014/US strain clustered with that of GII.3 strains, neither bootstrap values nor genetic distances reached values (>70% or >0.143, respectively) that enabled them to be classified within any known P genotype (*6*,*12*). The other GII.17 cluster C strains grouped with the Hu/GII.17/Gaithersburg/2014/US strain in the polymerase region and displayed only 1 amino acid substitution, confirming their similar evolutionary origins (Figures 1, 2). Of note, certain GII.17 viruses (probably cluster B) with the GII.P13 genotype in their polymerase circulated during 2004–2009 in various countries around the world (Technical Appendix). Full-length sequences of these GII.P13/GII.17 strains will be needed for determination of their evolutionary relationship with the new GII.17 cluster C viruses.

## Conclusions

Surveillance in China has shown that norovirus GII.17 predominated over GII.4 Sydney as the cause of outbreaks during the 2014–15 season (*9*). The sequences reported in that study showed a high similarity with the Hu/GII.17/Gaithersburg/2014/US strain and clustered within GII.17 cluster C. Thus, we conclude that the same GII.17 virus that caused outbreaks in Asia was present in the United States near the beginning of the 2014–15 season. It is possible that these new GII.17 viruses bear substitutions in VP1 that define new antigenic sites, which, coupled with new characteristics in ORF1, provide an adaptive advantage for rapid spread. Continued monitoring of emerging norovirus strains is needed for a better understanding of their evolution and epidemiology.

**Technical Appendix.** Differences in the major capsid protein among GII.7 noroviruses, phylogenetic relationships among GII.17 noroviruses circulating worldwide, and relationship of Hu/GII.17/Gaithersburg/2014/US with other GII.17 noroviruses.
