# Supplementary material for: Genome of Emerging Norovirus GII.17, United States, 2014
Source: Emerg Infect Dis. 2015 Aug;21(8):1477–9. doi: 10.3201/eid2108.150652 (PMC4517714; doi:10.3201/eid2108.150652)
Supplement: Supplementary file 1 — Technical Appendix. Differences in the major capsid protein among GII.7 noroviruses, phylogenetic relationships among GII.17 noroviruses circulating worldwide, and relationship of Hu/GII.17/Gaithersburg/2014/US with other GII.17 noroviruses. [file 15-0652-Techapp-s1.pdf]

DOI: http://dx.doi.org/10.3201/eid2108.150652

Genome of Emerging Norovirus GII.17, United States, 2014

Technical Appendix

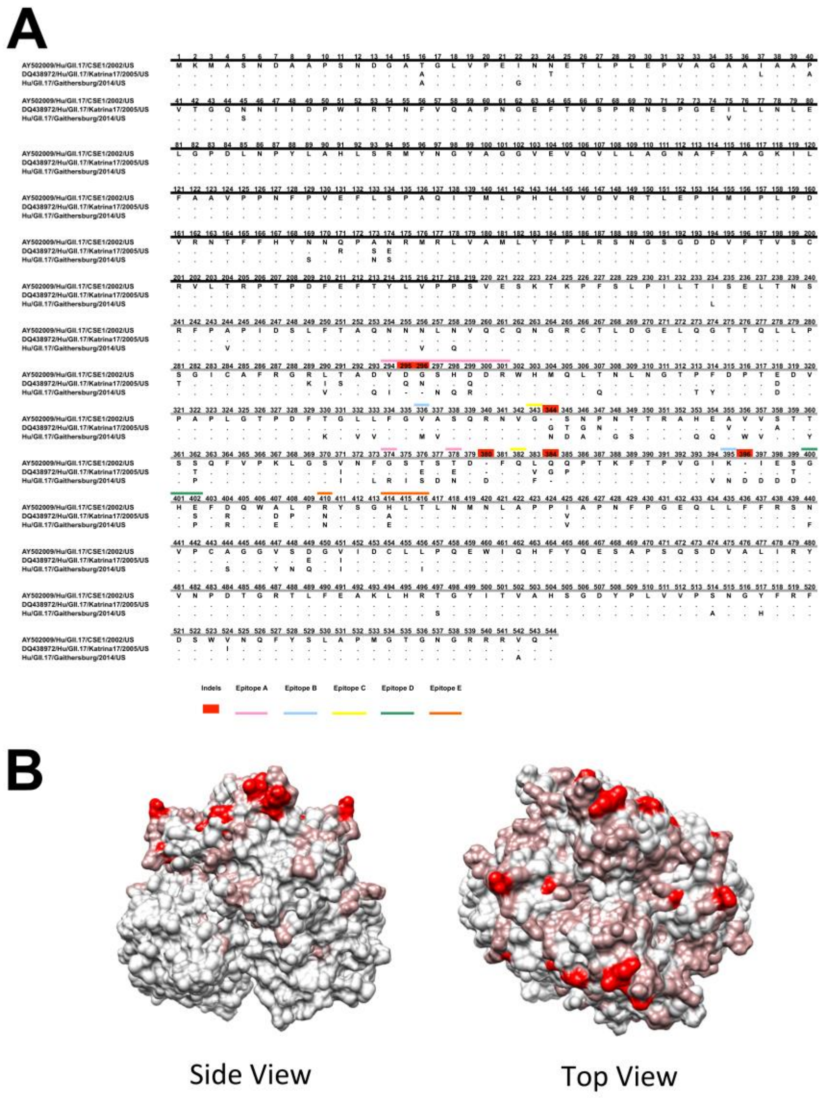

Technical Appendix Figure 1. Differences in the major capsid protein (VP) among GII.7 noroviruses. A) Amino acid sequence alignment of the VP1 sequences from 1 representative strain from each of the 3 GII.17 clusters described. The shell (S) domain is highlighted with a dark line, and the protruding (P) domain is highlighted with a gray line. The color code for each of the GII.4 norovirus epitopes and

insertions is indicated. B) Top and side views of the P domain of a model of the Hu/GII.17/Gaithersburg/2014/US strain comparing the locations of the substitutions from cluster C strains and the cluster A and B strains. Substitutions are shown in brown, and indels (insertion or deletions) are shown in red. Modeling was done with I-Tasser (*1*) and visualized with Chimera (*2*).

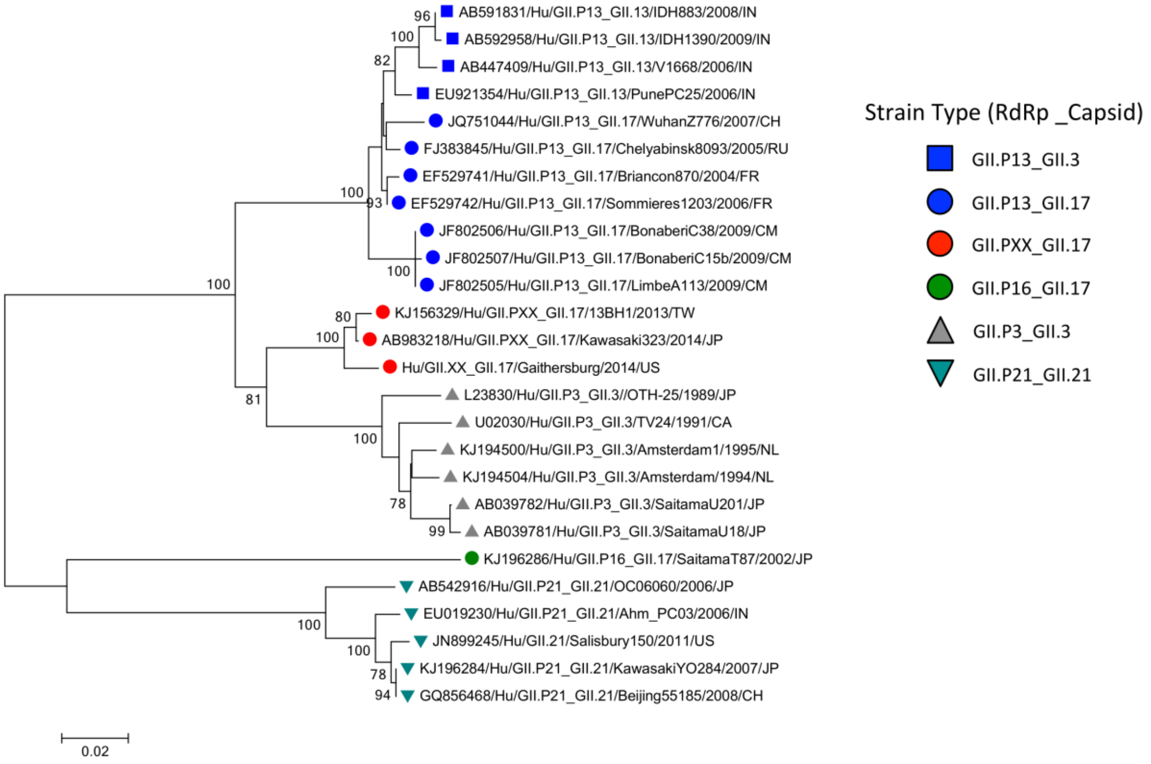

Technical Appendix Figure 2. Phylogenetic relationships among GII.17 noroviruses circulating worldwide. Phylogenetic tree of the RdRp region from novel GII.17 strains (red circle) and strains from genotypes GII.P3 (gray triangle), GII.P13 (blue square), GII.P16 (green circle), and GII.P21 (teal inverted triangle). Recent circulating GII.P13/GII.17 strains are represented by blue circles. Phylogenetic analyses were conducted by using MEGA version 6 (*3*), neighbor joining as the algorithm for reconstruction, and Tamura-Nei as the model of substitution. Bootstrap (500 replicates) analysis was used for the statistical support of the tree, values >70% are shown. Scale bar indicates nucleotide substitutions per site.

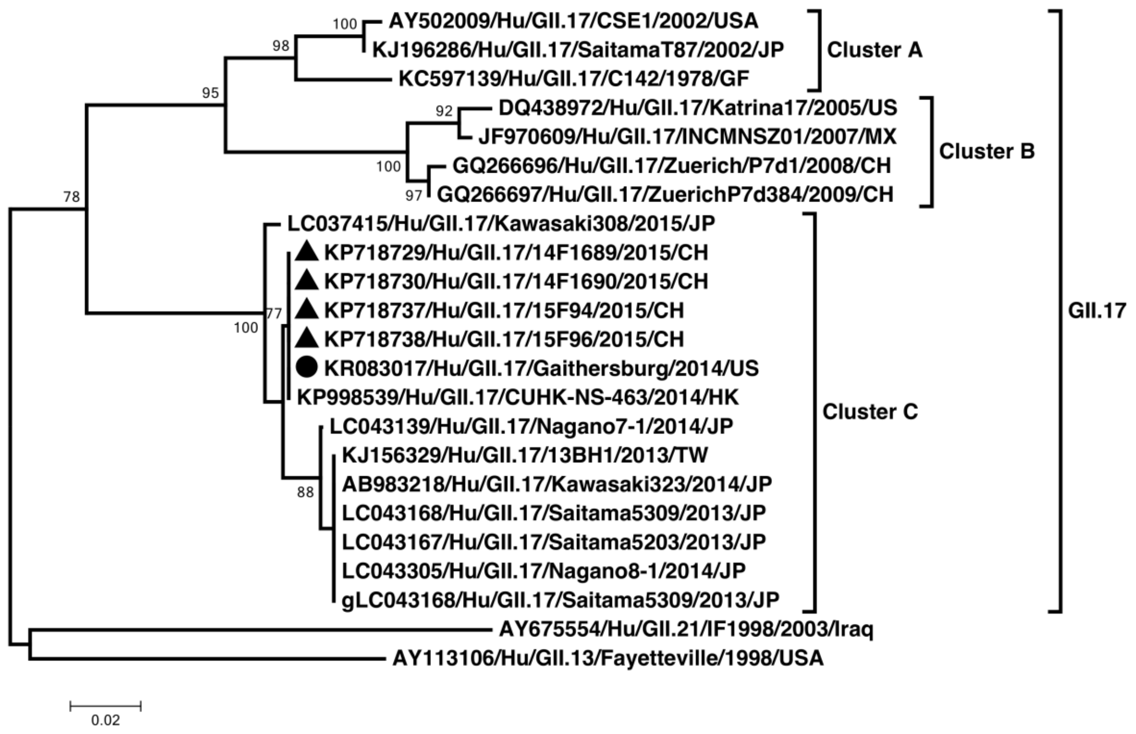

Technical Appendix Figure 3. Relationship of Hu/GII.17/Gaithersburg/2014/US with other GII.17 noroviruses. Phylogenetic tree of the Hu/GII.17/Gaithersburg/2014/US N-terminal region of VP1 region in comparison with those of GII.17 norovirus strains available in public genetic databases, including those causing large outbreaks in the Guangdong Province, China. Phylogenetic analyses were conducted by using MEGA version 6 (*3*), neighbor joining as the algorithm for reconstruction, and Tamura-Nei as the model of substitution. Bootstrap (500 replicates) analysis was used for the statistical support of the tree, values >70% are shown. The Hu/GII.17/Gaithersburg/2014/US strain is indicated by a filled circle and those from Guangdong Province, China, by a filled triangle. Scale bar indicates nucleotide substitutions per site.
